# Viral infection, APOBEC3 dysregulation, and cancer

**DOI:** 10.3389/fgene.2024.1489324

**Published:** 2024-12-23

**Authors:** Jake Lehle, Mohadeseh Soleimanpour, Samira Mokhtari, Diako Ebrahimi

**Affiliations:** ^1^ Host-Pathogen Interaction Program, Texas Biomedical Research Institute, San Antonio, TX, United States; ^2^ Department of Microbiology, Immunology, and Molecular Genetics, University of Texas Health San Antonio, San Antonio, TX, United States; ^3^ Department Molecular Microbiology and Immunology, The University of Texas at San Antonio, San Antonio, TX, United States

**Keywords:** APOBEC3, viral infection, cancer, HPV, HBV, EBV, HPyV

## Abstract

Viral infection plays a significant role in the development and progression of many cancers. Certain viruses, such as Human Papillomavirus (HPV), Epstein-Barr Virus (EBV), and Hepatitis B and C viruses (HBV, HCV), are well-known for their oncogenic potential. These viruses can dysregulate specific molecular and cellular processes through complex interactions with host cellular mechanisms. One such interaction involves a family of DNA mutators known as APOBEC3 (Apolipoprotein B mRNA Editing Catalytic Polypeptide-like 3). The primary function of these cytidine deaminases is to provide protection against viral infections by inducing viral mutagenesis. However, induction and dysregulation of A3 enzymes, driven by viral infection, can inadvertently lead to cellular DNA tumorigenesis. This review focuses on the current knowledge regarding the interplay between viral infection, A3 dysregulation, and cancer, highlighting the molecular mechanisms underlying this relationship.

## 1 Introduction

Research into the association between viral infections and cancer in humans began in the 1960s with the discovery of the Epstein-Barr virus (EBV) in cells from Burkitt’s lymphoma patients (Epstein et al., 1964). Current estimates suggest that each year between 13% and 18% of all new cancer cases are caused by infection (Pisani et al., 1997; Parkin, 2006; De Martel et al., 2012; Plummer et al., 2016; de Martel et al., 2020). Viruses can contribute to cancer initiation and progression through several mechanisms, including viral insertion into the host genome, which disrupts cell cycle regulation genes (Hayward et al., 1981), proviral integration of oncogenes into the viral genome (Duesberg and Vogt, 1970), and persistent infections that lead to chronic inflammation, dysregulating normal immune function and turning it against the host (Emanuele Liardo et al., 2021).

One of the key mechanisms dysregulated by viral infection in cancer involves a family of cytidine deaminases known as apolipoprotein B mRNA editing enzyme catalytic polypeptide-like 3 (APOBEC3 or A3). These enzymes are essential components of the host’s innate immune system, and their primary function is to protect the host cells from exogenous viruses (Doehle et al., 2005; Peng et al., 2007; Vartanian et al., 2008; Ooms et al., 2012) and endogenous elements (Stenglein and Harris, 2006; Vartanian et al., 2008; Horn et al., 2014) through mutation-dependent (Maciejowski et al., 2020) and mutation-independent mechanisms (Hakata and Miyazawa, 2020). However, certain viral infections and other tumor-initiating events can result in a non-specific targeting of host DNA by these proteins. Indeed, A3-induced mutational signatures SBS2 and SBS13 (Single base substitution profiles 2 and 13) are present in more than 50% of tumor genomes and are usually associated with adverse outcomes (Alexandrov et al., 2020; Yaacov et al., 2024). The mutational signatures SBS2 and SBS13 are characterized by C-to-T and C-to-G mutations within the TCT and TCA trinucleotide motifs and are attributed particularly to dysregulation of A3A, A3B, and, to a lesser extent, A3H haplotype I (Starrett et al., 2016; Olson et al., 2018; Petljak et al., 2022; Durfee et al., 2023).

With respect to the role of A3 enzymes in cancer, these proteins can be classified into two groups. The first group includes A3A and A3B, which are dysregulated in tumor cells and have been shown to be the primary sources of SBS2 and SBS13 in many tumors (Beale et al., 2004; Warren et al., 2017). Group two includes A3C, A3D, A3F, A3G, and A3H, which often express in immune cells and some normal non-immune cells and are not associated with tumor mutagenesis (Hultquist et al., 2011; Jang et al., 2024). It is important to note that some A3s in this group such as A3G and certain haplotypes of A3H are potent DNA mutators, but they almost exclusively mutate viral DNA as an antiviral defense mechanism, and do not catalyze host DNA.

Here, we provide an overview of the interplay between viral infection, A3 dysregulation, and tumor mutagenesis and progression. We start by an introduction to the biology of A3 enzymes, highlighting their structural features, substrate binding, localization, and overall function. Next, we explore the connection between viral infections and the dysregulation of A3 enzymes, with contribution to the development and progression of cancers. Finally, we catalog various viruses and their associations with dysregulated molecular pathways across cancers and summarize current gaps in knowledge and future directions in the field.

### 1.1 A3 sequence and structural features

**FIGURE 1:**
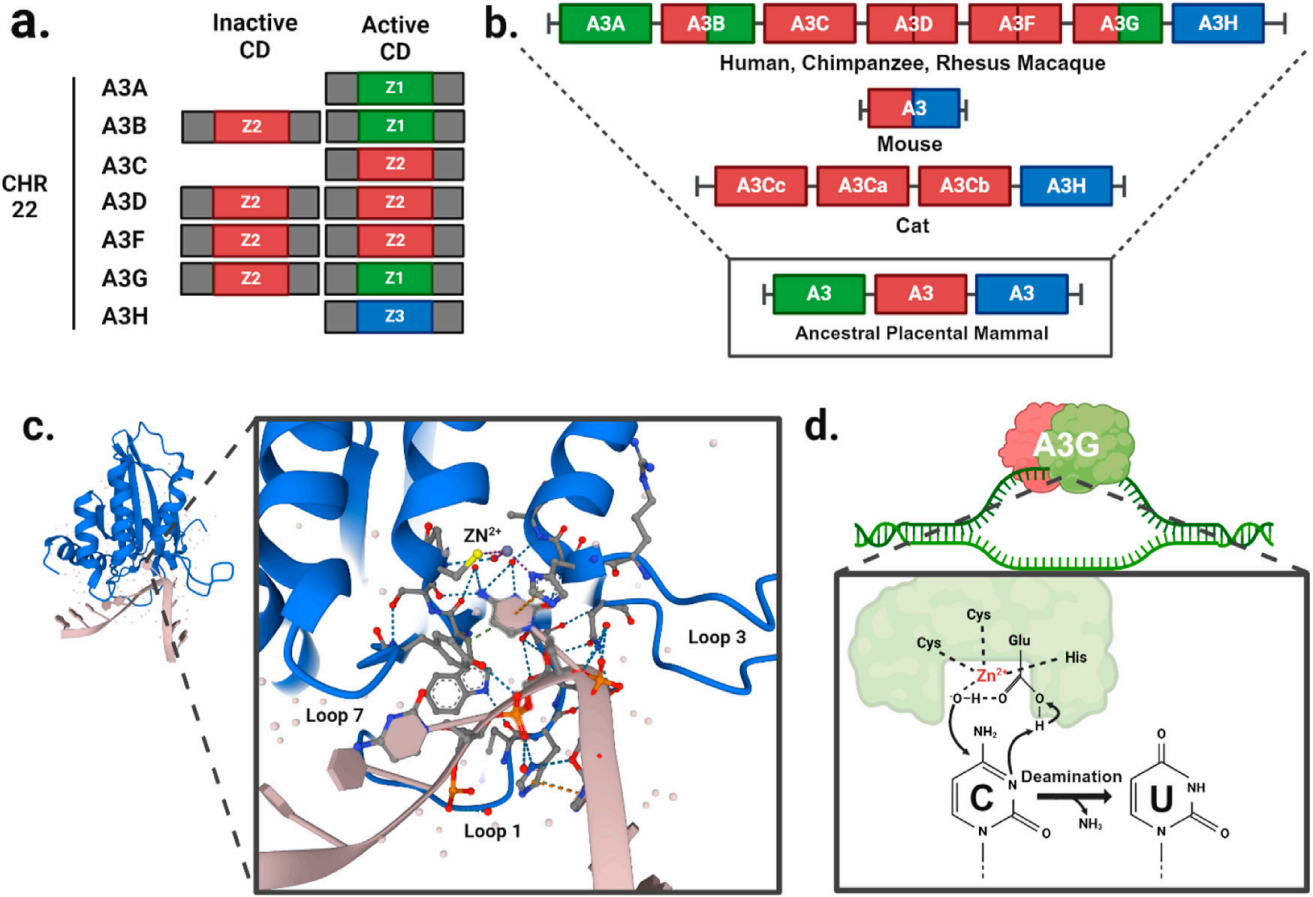
A3 function and evolution. **(A)** Gene duplication events have given rise to A3A/C/H with a single Z-domains and A3B/D/F/G with two Z-domains in human. **(B)** The evolutionary divergence of the A3 gene composition in present-day mammals traces back from humans and primates to a likely ancestral placental mammal. **(C)** Crystal structure of A3G cytidine deaminase domain with substrate viral cDNA (PBD: 6BUX). The inset shows the interaction of the three loops (1, 3, 7) within the substrate binding groove, and a zinc atom (yellow) with the cytosine nucleotide of the viral cDNA. **(D)** Schematic of cytidine deamination by a direct nucleophilic attack on the C4 pyrimidine ring. In the catalytic site, cysteines and histidine coordinate a zinc atom (Zn2+) to hydrolyze a water molecule donating a proton to the catalytic glutamate and forming a hydroxyl group. The glutamate acts as a proton shuttle during the catalysis, converting deoxycytidines to deoxyuridines.

Since the initial discovery of *A3G* (Jarmuz et al., 2002), seven A3 family members have been identified in humans, located on chromosome 22 (Figure 1A). Comparative genomic studies suggest a rapid evolution of the *A3* loci across mammals as evidenced by high rates of amino acid substitutions and gene duplication events (Uriu et al., 2021). The number of A3 copies varies significantly among branches of the mammalian phylogenetic tree (Figure 1B). It is believed that this diversity originated from a common ancestral placental mammal, which possessed three tandem copies of *A3* in a head-to-tail arrangement. This organization enabled rapid evolutionary diversification through unequal cross over events between species, resulting in gene expansions in some lineages and contractions in others. As a result, humans, great apes, and Old-World monkeys have seven A3 enzymes (Sawyer et al., 2004), while mammals such as domesticated cats have four *A3* gene variants (Münk et al., 2008; Troyer et al., 2019), and mice have only a single *A3* gene (Zhao et al., 2020; Tsukimoto et al., 2022).

*A3* genes express either single- or double-domain proteins, with each family member having at least one catalytic domain (Figures 1A, B). The A3 catalytic domains are classified into three types of Z1, Z2, and Z3 based on the amino acid sequences of the zinc-finger domain (LaRue et al., 2009). The catalytic site is characterized by a histidine and a single glutamate in an HxE motif as well as two cysteines in a CPx2-4C motif, each located at the ends of two neighboring conserved α-helices (Figure 1C). These conserved motifs are essential for deamination function, which occurs via zinc (Zn)-mediated hydrolysis of the 4-NH2 group on ssDNA cytosine residues. In this process, water molecules are deprotonated to form a catalytic glutamate (Glu) that acts as a general acid/base in the deamination mechanism (Figure 1D). The Zn-stabilized hydroxide ion attacks the C4 position of the ssDNA cytosine, converting it to uracil by forming a C4-O olefin and releasing an ammonia molecule (NH3).

### 1.2 A3 expression and localization

Each A3 member has a unique cellular and subcellular localization that contributes to defining their role in the innate immune system (Table 1). All A3 members are expressed at various levels in hematopoietic and lymphoid tissues (Table 1). As a result, tissues with the highest expression of A3 are primarily associated with the lymphatic system (e.g., peripheral blood, bone marrow, and lymph nodes). The specific cell types within these tissues expressing A3 members include B cells (both naïve and memory), CD4^+^ and CD8^+^ T cells, NK cells, dendritic cells, and macrophages. While most cell types expressing A3 enzymes are immune cells, other cell types including epithelial, endothelial, and fibroblasts also express members of the A3 enzyme family such as A3B, A3C, and A3G (Lin et al., 2009; Okeoma et al., 2010; Pautasso et al., 2018) and expression can be found in highly perfused organs with epithelial linings such as the intestine, bladder, respiratory tract, and liver.

**TABLE 1:** Localization and expression of A3 family members in normal and cancer tissues.

Protein	Localization	Normal cell/tissue expression (Thul and Lindskog, 2018)	Tumor tissue expression (Butler and Banday, 2023)
A3A	Nuclear/Cytoplasmic	Cells: Monocytes and macrophages Tissues: Lymphoid tissue, bone marrow, and urinary bladder	Multiple adrenal, bile duct, bladder, breast, leukemia, penile
A3B	Nuclear	Cells: Plasma, ductal, erythroid, and pancreatic endocrine cells Tissues: Lymphoid tissue, bone marrow, urinary bladder, intestine, and kidney	Multiple adrenal, B-cell lymphoma, bile duct, bladder, breast, cervical, esophageal, gastric, glioma, head and neck, kidney, lung, nasopharyngeal, ovarian, prostate, skin, uterus
A3C	Nuclear/Cytoplasmic	Cells: Macrophages, NK cells, Eosinophils adipose progenitor cells, skeletal muscle, fibroblasts, Sertoli cells Tissues: Peripheral blood cells, lymphoid tissue, bone marrow, skin, muscle, breast tissue, reproductive system, kidney, urinary bladder, gastrointestinal tract, liver, gallbladder, intestines, respiratory tract, and endocrine tissues	B-cell lymphoma, glioma
A3D	Cytoplasmic	Cells: T cells, B cells, plasma, and NK cells Tissues: Peripheral blood cells, lymphoid tissue, and bone marrow	Glioma
A3F	Cytoplasmic	Cells: Macrophages Tissues: Peripheral blood cells, lymphoid tissue, bone marrow, reproductive system, and endocrine tissues	Multiple adrenal, glioma
A3G	Cytoplasmic	Cells: T cells, NK cells, and plasma cells Tissues: Peripheral blood cells, lymphoid tissue and bone marrow, breast tissue, reproductive system, urinary tract, bladder, gastrointestinal tract, liver, gallbladder, and respiratory tract	Multiple adrenal, glioma, pancreatic
A3H	Nuclear/Cytoplasmic	Cells: T cells and NK cells Tissues: Peripheral blood cells, lymphoid tissue, and bone marrow	Breast, glioma

A3 family members A3A, A3C, and A3H are expressed in both the cytoplasm and nucleus during interphase (Lackey et al., 2013; Cheng et al., 2021) (Table 1). Given that these are single domain proteins (Figure 1A) with sizes <25 kDa, this localization is likely due to passive diffusion through nuclear pores allowing entry into and exit from the nucleus (Salamango et al., 2018). A3B, in contrast, is a double-domain protein with a size of >50 kDa (Lackey et al., 2012) and is located in the nucleus due to its N-terminal nuclear localization signal (NLS) (Salamango et al., 2018). Thus, A3B is sequestered in the nucleus and is sterically too large to diffuse out (Lackey et al., 2012; Salamango et al., 2018). By contrast, other double-domain A3 enzymes (A3D, A3F, and A3G) lack an NLS sequence and are restricted to the cytoplasm (Lackey et al., 2013). Notably, A3G even contains a cytoplasmic retention signal, further solidifying its subcellular localization in the cytoplasm. During mitosis, all A3 members are excluded from chromatin structures (Lackey et al., 2013) presumably to limit any A3-driven DNA mutagenesis. However, the highly condensed chromatin state, which lacks an abundance of transcription bubble ssDNA structures, is likely at low risk of mutagenesis (Zhang et al., 2019).

### 1.3 A3-induced viral inhibition and viral counter-defense mechanisms

The antiviral function of A3 enzymes is mediated through both deaminase-dependent and deaminase-independent mechanisms. Differences in sequence preference can lead to large differences in the functionality of A3 family members. The strong preference for deamination of CC dinucleotides by A3G on the minus strand of retroviral cDNA during reverse transcription leads to GG > AG mutations in the viral genome, with the potential to convert TGG (tryptophan codon) to TAG (stop codon). In contrast, A3D/F/H preferentially target TC sequences resulting in GA > AA mutations which can convert TGG into TGA (stop codon) only if the tryptophan codons are followed by an adenine base (TGGA > TGAA). Tryptophan plays a critical role in stabilizing, anchoring, and orienting lipid bilayers and capsid structural proteins, making it highly conserved across many viral genomes. Therefore, it represents an Achilles’ heel for targeting a wide range of viruses (Villanova et al., 2020).

However, deaminase-independent functions, which do not lead to any change in the base composition of the viral genome, also serve as an effective antiviral mechanism. A3 enzymes associate with viral RNA within the infected cell, impacting various aspects of reverse transcription, including inhibiting reverse transcriptase binding (Chaurasiya et al., 2014; Morse et al., 2017), tRNA annealing (Guo et al., 2006; Guo et al., 2007), and template switching (Adolph et al., 2019). In addition, A3s can interfere with viral integration into the host genome by directly impacting viral integrase and DNA end processing (Luo et al., 2007; Mbisa et al., 2010).

**FIGURE 2:**
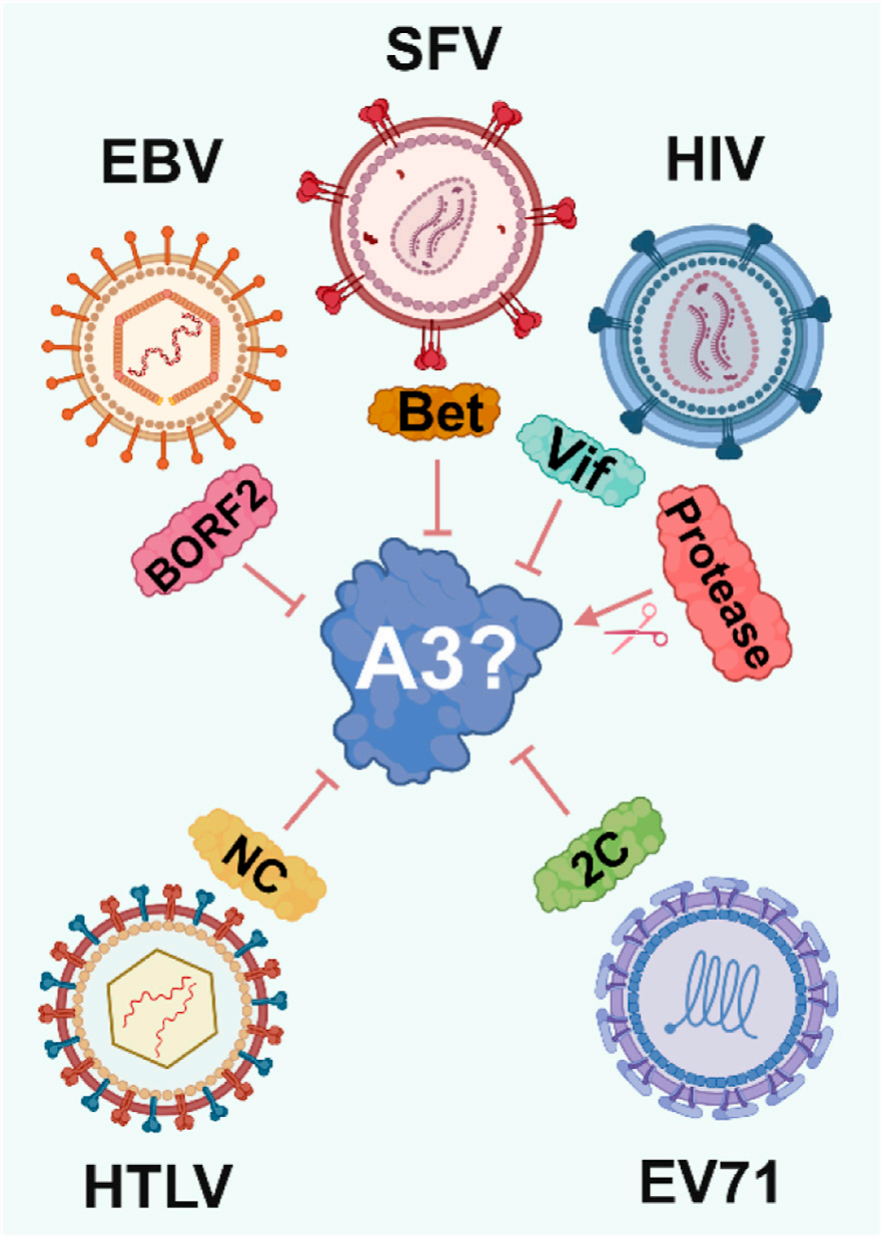
Viral strategies to evade A3 enzymes. To avoid A3-induced viral inhibition viruses have independently evolved proteins that target A3 through various mechanisms, ranging from ubiquitination and proteasomal degradation by HIV-1 Vif to altering A3 localization by EBV BORF2.

Viruses have evolved diverse strategies to counteract A3 enzymes. Notably, these include proteins such as Vif and Vpr in HIV-1, RdRp (Okumura et al., 2008) and 2C in Enterovirus 71 (EV71) (Yuan et al., 2018), Bet in simian foamy viruses (SFV) (Vasudevan et al., 2021), and NC in Human T-lymphotropic virus 1 (HTLV) (Derse et al., 2007) (Figure 2). Strikingly, each of these mechanisms has evolved independently, preventing lethal levels of A3-induced mutations in viral genomes and mitigating other mutation-independent actions of A3 enzymes.

### 1.4 The link between viral infection, A3 dysregulation, and oncogenesis

**FIGURE 3:**
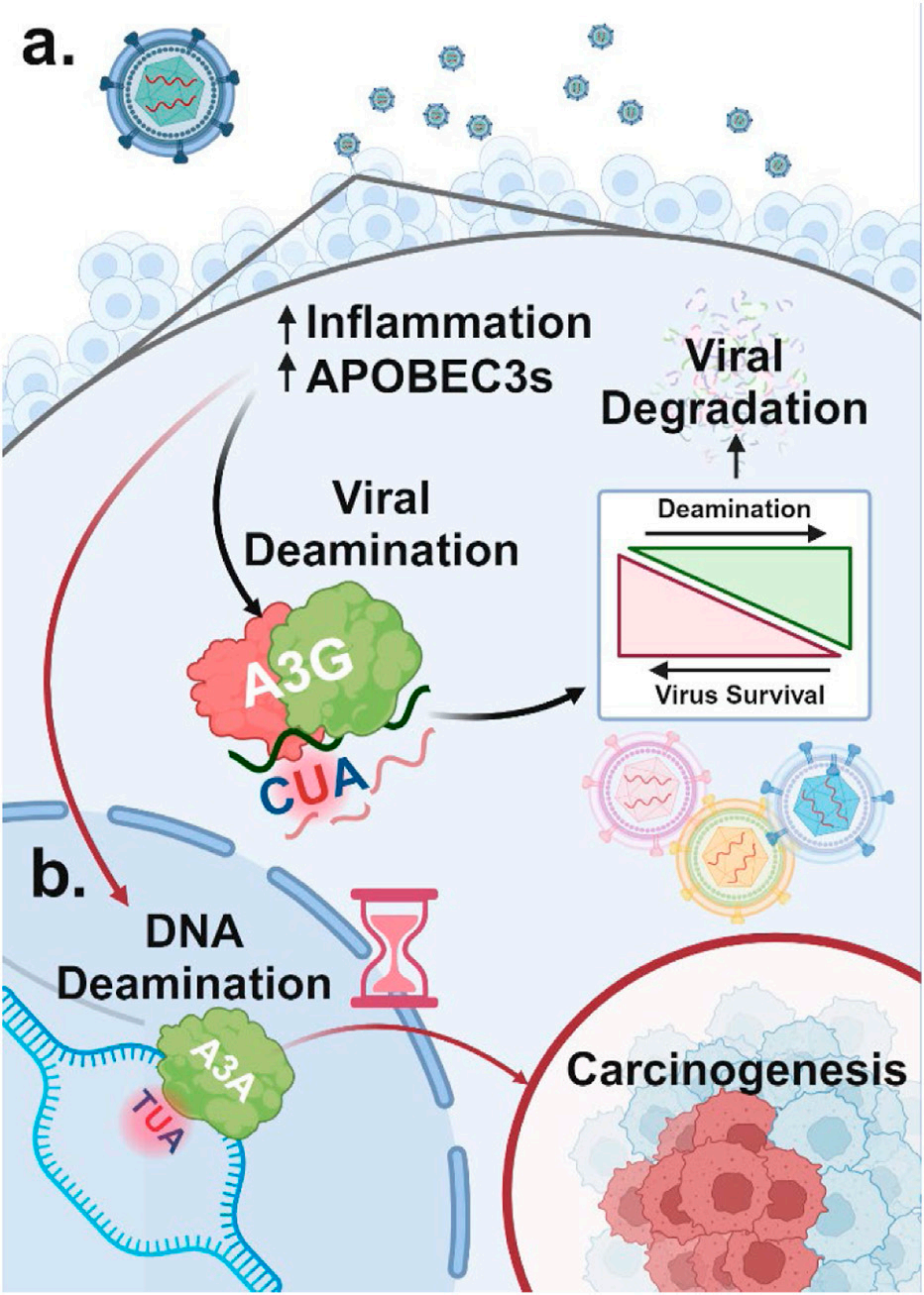
Dual role of A3 proteins in viral restriction and oncogenesis **(A)** A3 proteins are upregulated in response to viral infection due to increased inflammation. Viral DNA regions that become single-stranded can undergo A3-mediated deamination, which, at high levels, can degrade the viral genome and restrict viral replication. In contrast, lower levels of A3-induced mutations that do not lead to viral restriction can promote viral diversification and evolution. **(B)** A3 enzymes upregulated in response to viral infection can also target host single-stranded DNA for cytidine deamination. This results in C-to-U mutations, potentially causing significant DNA damage and genome instability, which may contribute to carcinogenesis.

Regulatory mechanisms controlling the expression of each A3 enzyme remain an active area of research. However, numerous stimuli that increase A3 expression as part of the innate immune response have been identified (Chen et al., 2006; Mehta et al., 2012). These stimuli include activation of Toll-like receptors (TLR3, TLR4, and TLR7) by viral particles (Wang et al., 2011) and type I interferon (IFN) signaling triggered by inflammation (Peng et al., 2006). This establishes a clear link between viral infections and the dysregulation of normal A3 enzymes as an innate immune system response. Indeed, A3 enzymes are upregulated following viral infection (Milewska et al., 2018) (Figure 3A). For example, retroviral reverse transcripts that evade A3 inhibition can be further targeted by cyclic GMP-AMP synthase, which binds and activates the STING protein, thereby increasing the expression of IFNs and other cytokines (Lahaye et al., 2013), raising A3A levels and reducing retroviral reverse transcription (Decout et al., 2021). However, to prevent a prolonged inflammatory response and tissue damage and restore the normal expression of A3 enzymes following inflammatory signals, A3A has been shown to compete for IFN-stimulated regulatory elements, inhibiting IFN-stimulated genes, in a negative feedback loop (Taura et al., 2022).

While A3 enzymes are effective defenders against viral threats, they can also target host ssDNA, potentially leading to widespread deamination. This damage is primarily driven by A3A and to a lesser extent A3B due to their high deaminase activity and access to the nuclear compartment (Figure 3B) (Chan et al., 2015; Cortez et al., 2019). Although deamination of host DNA can occur anywhere decondensed DNA becomes single-stranded due to “DNA breathing,” it is more likely to occur at replication forks, particularly on the lagging strand, which has higher single-strand exposure (Roberts et al., 2012; Green et al., 2016; Hoopes et al., 2016; Seplyarskiy et al., 2016).

The molecular consequences of genomic ssDNA deamination are usually repaired by base excision repair (BER) mechanisms, the primary repair mechanism for small, non-helix-distorting, base lesions like A3-induced mutations (Wilson and Bohr, 2007). The repair process starts with uracil-DNA glycosylase (UNG, a.k.a UDG) recognizing the uracil base introduced by A3 enzymes. UDG removes the uracil base by cleaving the bond between the uracil and the deoxyribose sugar, creating an abasic site (a.k.a. AP site). The AP site is then processed by an AP endonuclease (APE1), which cuts the DNA backbone at this position, generating a single-strand break. DNA polymerase β (Pol β) subsequently fills the gap by inserting the cytosine nucleotide, and DNA ligase seals the nick.

Although BER is often an effective process, excessive numbers of mutations induced by A3 enzymes can overwhelm the BER pathway, leaving many lesions nonrepaired. Additionally, Pol β or other polymerases can sometimes misincorporate incorrect nucleotides, especially when dNTP levels are distorted. This misincorporation can lead to additional mutations. Furthermore, if the single-strand breaks (SSBs) generated by BER are not quickly repaired or if they occur near other lesions, they can convert into double-strand breaks (DSBs), resulting in larger chromosomal rearrangements. Additionally, APOBEC enzymes often produce clustered mutations (a.k.a. kataegis) (Nik-Zainal et al., 2012). BER is often ineffective at fully resolving these clustered mutations (Elango et al., 2019).

Certain chronic viral infections that result in persistently high A3 levels, combined with other tumor-initiating events, can intensify the non-specific targeting of host DNA by these enzymes, thereby driving oncogenesis (Figure 3B). Numerous viral infections have been associated with the development and progression of specific cancers. However, for many other viruses lacking an obvious association, further investigation and relative quantification of how each virus dysregulates A3 function could provide valuable insights into their oncogenic potential. In the remainder of this review, we will focus on specific examples of such infections, demonstrating how long-term shifts in the balanced expression of A3 contribute to the development of various cancer types.

## 2 Viruses

### 2.1 Human papillomavirus (HPV)

#### 2.1.1 HPV overview

HPVs are double-stranded DNA viruses with a circular genome of ∼8 kb. These viruses do not have an envelope, and their genomes are enclosed within a protein capsid (Graham, 2017). There are more than 200 known HPV types, which are categorized into two major groups of “low risk” and “high risk.” The former group (e.g., HPV6 and HPV11) is often associated with benign conditions such as warts, while the latter (e.g., HPV16 and HPV18) are strongly linked to cancers in the cervix, vulva, vagina, penis, anus, and head and neck (Chen et al., 2017; Yan et al., 2020).

HPV infection and its associated cancers remain a significant global health issue. Studies estimate that 90% of cervical and anal cancers, 70% of oropharyngeal cancers, 70% of vulvar and vaginal cancers, and 60% of penile cancers are associated with infection by high-risk HPV (Heller et al., 2018). Importantly, HPV prevalence is notably higher in Oceania and Africa compared to Europe and Asia, with the highest prevalence observed in low- and middle-income regions (Bruni et al., 2023). Furthermore, the rate of HPV infection is rising in men (Formana et al., 2012; Bruni et al., 2023).

**FIGURE 4:**
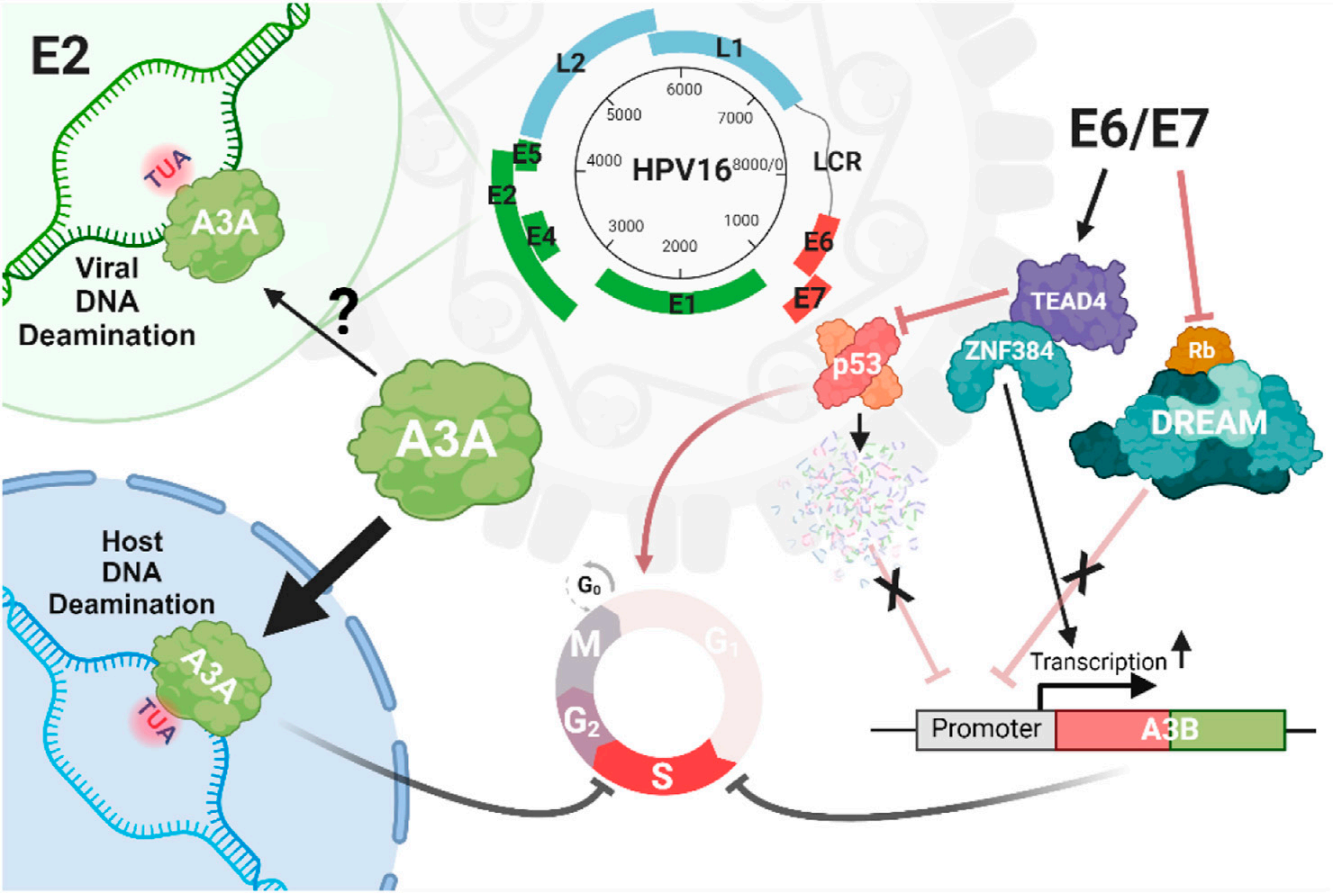
Interplay between A3 and HPV. There is limited and conflicting evidence for deamination of the HPV by A3, except for the *E2* locus, which has been reported to be enriched for mutations within the motifs preferentially targeted by A3 enzymes. However, there are compelling evidence for the A3A-induced mutation of host genomes driven by infection with high-risk HPV strains such as HPV16. HPV can promote genomic damage through the expression of oncoproteins E6/E7, which upregulate *A3* genes such as *A3B* by eliminating the inhibitory impacts of p53 and Rb cell death proteins, or through the interaction of the zinc finger protein ZNF384 and TEAD4 on the proximal (+1/+45 bp) *A3B* promoter.

HPVs infect keratinocytes in the basal layer of cutaneous or mucosal epithelia, leading to their differentiation and migration toward the skin surface (Kranjec et al., 2017; Faden et al., 2021). This virus family exploits the DNA replication machinery of the host cells, driving the infected cells into their S-phase to produce viral proteins. The E6 and E7 oncoproteins of high-risk HPV types play a critical role in this process (Figure 4). Specifically, E7 binds to and degrades the retinoblastoma protein (pRB), resulting in the expression of genes involved in DNA synthesis mediated by E2F transcription factors. Additionally, E6 drives p53 degradation, resulting in cell cycle arrest or apoptosis triggered by DNA damage (Riva et al., 2019; Raina MacIntyre et al., 2021).

The E6 and E7 proteins are expressed during productive HPV infection, however, their expression is typically regulated by the viral E2 protein to ensure a controlled viral life cycle. By contrast, in high-risk HPV infections, the break on the expression of E6 and E7 is removed, resulting in their persistent high expression, uncontrolled cellular proliferation, and inhibition of cellular differentiation. This drives the progression of cervical intraepithelial neoplasia (CIN) from low-grade lesions (CIN1) to high-grade lesions (CIN2 and CIN3), and consequently to invasive cervical cancer. Integration of high-risk HPV into the host DNA can lead to the disruption of E2, further elevating the expression levels of E6 and E7, driving malignant transformation (Liu et al., 2020; Raina MacIntyre et al., 2021).

#### 2.1.2 Interaction between HPV and A3 enzymes

##### 2.1.2.1 HPV oncoproteins and A3 expression

The life cycle of high-risk HPV types involves complex interactions between HPV oncoproteins and the host’s A3 enzymes (Figure 4). These interactions play a significant role in HPV pathogenesis and carcinogenesis, particularly in the mutation of host DNA by A3 enzymes (Sui et al., 2019). HPV oncoproteins, particularly E6 and E7 from high-risk HPV types, regulate the expression of *A3* genes, thus influencing the host immune system and viral persistence (Wallace and Münger, 2018; He et al., 2022; Trimmel et al., 2022). For instance, by disrupting the cullin-RING-based E3 ubiquitin ligase complexes, E7 can lead to a stable expression of *A3A* by inhibiting its degradation (Westrich et al., 2018). Additionally, E6 and E7 can increase the expression of *A3B* in keratinocytes through different mechanisms: E6 can recruit TEAD4 to both inhibit the tumor suppressor protein p53, which normally functions to transcriptionally suppress various genes, including *A3B* as well as bind to the *A3B* promoter increasing expression (Mori et al., 2015; Mori et al., 2017). The viral oncoprotein E7 disrupts the DREAM complex (DP1, RB-like, E2F4, and MuvB) by interacting with and degrading pRb family members, such as p107 and p130, which normally function to inhibit the E2F transcription factors. These factors regulate the expression of many genes including *A3B*. The upregulation of *A3B* is thought to occur due to E7-mediated degradation of pRb, which releases E2F and activates genes such as *A3B*. These regulatory mechanisms suggest that HPV oncoproteins promote viral persistence and potentially contribute to carcinogenesis by upregulating A3 enzymes and inducing host DNA mutagenesis (Doorbar et al., 2012; Pinidis et al., 2016; Conner et al., 2020; Chahoud et al., 2021).

##### 2.1.2.2 Inhibition of HPV by A3 enzymes

Evidence for the antiviral function of A3s in HPV is limited and sometimes inconsistent. Furthermore, unlike HIV-1, which is known to undergo significant hypermutation by A3 enzymes, HPV genomes show minimal, if any, A3-induced mutations (Hirose et al., 2018). Unlike HIV-1 and several other viruses, HPV does not seem to have evolved a mechanism to counteract A3 enzymes. Nevertheless, two reports have indicated that A3-induced mutations, may be selectively enriched in the E2 region of the genome (Wang et al., 2014; Faden et al., 2021). A study by Warren et al. provides evidence supporting the role of A3 enzymes in restricting HPV. The study shows that HPV virions packaged in cells overexpressing a catalytically active A3A are significantly less infectious in keratinocytes (Warren et al., 2014). However, this effect is not observed with A3B, A3C, or even catalytically inactive A3A, suggesting that A3A may be the only A3 enzyme capable of restricting HPV through a deaminase-dependent mechanism. However, somewhat contradictory, the study did not identify A3A-induced mutations in the genomic regions previously shown to be edited by A3 enzymes.

Another study in which an HPV16 pseudovirion production system was used, reports that A3A and A3C greatly reduce the infectivity of pseudovirions in HeLa cells. The study found that the expression level of A3A, but not A3C was negatively correlated with the number of encapsidated pseudovirions. On the other hand, A3C, but not A3A inhibited viral entry by binding to the HPV L1 capsid protein (Monjurul et al., 2015). Additionally, A3A-induced cell cycle arrest in HPV-infected cells has been proposed as an additional layer of indirect restriction against HPV replication. Discrepancies between these conflicting results could be due to differences in the HPV16 clone used and/or differences in the method used to measure infectivity across cell lines with potentially different regulation of A3B/A3C activity. These data highlight the need for studies to systematically investigate the restriction capacity of A3 enzymes against HPV16 across multiple cell types in order to clarify this conflict.

In the context of HPV infection, the role of A3 enzymes extends beyond simple viral restriction, as they primarily contribute to host DNA mutagenesis, evidenced by the enrichment of cancer mutational signatures SBS2 and SBS13 in HPV-associated tumors, particularly in cervical, bladder, and head and neck tumors (Chen et al., 2017; Qin et al., 2018; Smith and Fenton, 2019; Faden et al., 2021; Warren et al., 2022). These signatures are characterized by C-to-T transitions and C-to-G transversions within mostly TCA and TCT trinucleotide motifs (Zhu et al., 2020). The enrichment of A3 signatures SBS2 and SBS13 in these cancer genomes may suggest that HPV infection induces A3 activity, leading to increased mutagenesis. Notably, A3B, in particular, is consistently upregulated in HPV-positive cancers, further implicating this enzyme in the oncogenic process (Smith and Fenton, 2019; Chatfield-Reed et al., 2020). Importantly, A3-induced mutations have been shown to be responsible for many driver mutations in HPV-associated tumors. For instance, in nearly 15% of HPV-associated head and neck squamous cell carcinoma cases, A3-mediated mutations in key oncogenes like *PIK3CA* are observed, highlighting the role of A3 enzymes in driving genetic alterations that lead to cancer (Henderson et al., 2014; Riva et al., 2021).

#### 2.1.3 Nuclear factor kappa B (NF-κB) pathway activation

The NF-κB pathway plays a critical role in regulating immune responses, including A3 expression during HPV infection. This pathway, which is known to be activated in response to various stimuli, including viral infections, upregulates several A3 enzymes, particularly A3A and A3B (da Costa et al., 2016). In the case of *A3B*, the promoter region was previously shown to contain an activational binding site for p65/c-Rel heterodimers from the NF-κB protein family (Maruyama et al., 2016). While A3 upregulation by NF-κB is intended as a host’s antiviral response, this upregulation can sometimes contribute to tumor DNA mutagenesis observed in HPV-associated cancers (Senba et al., 2011). High levels of E6 and E7 oncoproteins, especially from high-risk HPV types, can downregulate the tumor suppressor RRAD, thereby inducing the NF-κB pathway (Gu et al., 2019), which leads to an elevated expression of A3 enzymes and increased tumor mutations. This dual role of the NF-κB pathway in HPV infection underscores the complexity of A3 responses to HPV infection and oncogenesis.

#### 2.1.4 Implications of A3 activity in HPV-Infected cells

##### 2.1.4.1 Host genomic instability and cancer progression

A3 dysregulation in HPV-infected cells significantly impacts genomic stability and cancer progression (Trimmel et al., 2022). A3 enzymes, particularly A3A and A3B, are potent DNA mutators that can lead to significant host genomic instability, driving the progression from pre-cancerous lesions to invasive cancers (Granadillo Rodríguez et al., 2020; Revathidevi et al., 2021). A critical feature of A3 enzymes is their ability to inflict a large number of mutations in tumor DNA, thereby increasing the probability of targeting regions of the genome essential for maintaining genomic stability. Indeed, several studies have shown that mutations in key genes such as *PIK3CA*, *FGFR*, *ERBB2*, and *PTEN* are likely caused by A3 enzymes and are frequently observed in HPV-positive cancers (Chahoud et al., 2021; Revathidevi et al., 2021; Trimmel et al., 2022). This feature contributes to the activation of oncogenic signaling pathways and further promotes cancer progression. The mutagenic effects of A3 enzymes are exacerbated by the persistent expression of E6 and E7 oncoproteins in HPV-infected cells, which disrupts normal cell cycle control and inhibits DNA repair pathways (Litwin et al., 2017; He et al., 2022). This creates a feedback loop in which A3 activity drives further genomic instability, leading to additional mutations and accelerating cancer development (Warren et al., 2022).

##### 2.1.4.2 A3 dysregulation, tumor mutagenesis, and disease outcome

The interplay between viral infection, A3 dysregulation, tumor mutagenesis, and disease outcomes has been addressed in a number of studies. For example, a study focusing on urothelial carcinoma indicated that the A3-induced mutational signature is common in upper urinary tract urothelial carcinoma (UTUC). The study showed that HPV E6 expression is positively associated with A3B expression, and both are correlated with favorable prognostic factors in UTUC, such as stage I and low-grade tumors. Moreover, high expressions of HPV E6 and A3B were shown to be linked to better disease-free survival in patients, suggesting a complex interplay that may differ from other HPV-associated cancers (He et al., 2022).

Research into squamous cell carcinoma of the penis (PSC), a rare malignancy, has also revealed a potential role for the A3 family in PSC progression, particularly in cases associated with HPV. A study examining 50 PSC patients found that lower expression levels of A3A, A3B, and A3C were associated with advanced PSC stages (Trimmel et al., 2022). Furthermore, HPV-positive PSC samples exhibited higher expression of A3 enzymes compared to HPV-negative samples. Although initial analyses suggested a link between A3 expression and disease-free survival, further analyses did not confirm this association, indicating the need for more extensive research to understand the role of A3 in PSC.

Another study focusing on A3A expression in penile squamous cell carcinoma (SCC) showed distinct differences between HPV-positive and HPV-negative tumors (Heller et al., 2018). In tumors lacking HPV, there was a notable reduction in A3A expression, especially in the more invasive areas, suggesting that A3A might play a tumor-suppressive role, whereas HPV-positive tumors consistently showed high levels of A3A throughout their malignant progression. These observations suggest that HPV infection may facilitate an environment that supports elevated A3A levels, in contrast to HPV-negative tumors where there is a selective downregulation of A3A during tumor progression. Furthermore, the study indicated that tumors exhibiting higher A3A levels tend to have lower rates of cell proliferation compared to those with diminished expression. These results underscore a complex relationship between A3A expression, HPV status, and tumor dynamics in penile SCC (Heller et al., 2018).

Recent research has further expanded our understanding of HPV genomic dynamics, particularly through studies examining clade-specific characteristics (Løvestad et al., 2022). For instance, genomic variability and chromosomal integration was investigated in two distinct clades of carcinogenic HPV types, Alpha-7 and Alpha-9, using the TaME-seq protocol to sequence cervical cell samples infected with HPV31, HPV33, and HPV45, alongside earlier findings on HPV16 and HPV18. The results showed the presence of A3-induced mutational signatures in the Alpha-9 clade (comprising HPV16, HPV31, and HPV33) but not in the Alpha-7 clade (comprising HPV18 and HPV45). Additionally, HPV45 displayed a higher number of minor nucleotide variants (MNVs) compared to other types, and the Alpha-7 clade exhibited a higher frequency of chromosomal integration than Alpha-9, with integration frequency correlating with increased diagnostic severity within the Alpha-7 clade. These findings highlight critical differences in the molecular mechanisms driving cervical cancer across these high-risk HPV types.

Unlike A3A and A3B, which are highly implicated in HPV-associated mutagenesis and cancer progression, A3D, A3F, and A3G have been primarily studied in the context of retroviruses, like HIV-1, where they exhibit strong antiviral effects (Burns et al., 2013). These enzymes are considered less relevant to HPV for several reasons, including their subcellular localization, substrate specificity, and lower expression levels in the tissues commonly infected by HPV.

##### 2.1.4.3 HPV viral genome evolution

Studies indicate that, with respect to viruses such as HIV-1, A3 enzymes act like a double-edged sword. They can both hypermutate the viral genome, leading to its inactivation, or induce sublethal levels of mutations, facilitating viral evolution, immune evasion, and drug resistance (Chahoud et al., 2021). In contrast, in the case of HPV, the mutagenic impact of the A3 enzymes is minimal and there is little evidence for HPV hypermutation (Løvestad et al., 2022). It is therefore plausible to assume that any mutagenic activity of A3 enzymes can increase the HPV genetic diversity, generating variants capable of evading immune detection and persisting within the host (Litwin et al., 2017). Indeed, modulation of A3 expression by the HPV oncoproteins, particularly E6 and E7, through pathways like NF-κB and interferon signaling could be a viral strategy to adapt to changing host environments (Liu et al., 2020).

To provide evidence for this adaptation strategy, studies have investigated the representation of short sequence motifs arguing that an A3 pressure over a long evolutionary timeframe would lead to a lower representation of A3 target motifs (Warren et al., 2015). There is indeed evidence for the depletion of TC dinucleotides in the genomes of high-risk alpha-papillomaviruses, such as HPV16 and HPV18 (Poulain et al., 2020). This depletion is thought to result from A3-mediated editing, which has selected viral variants less susceptible to A3 activity.

In summary, the interaction between HPV and A3 enzymes reflects a complex interplay between viral pathogenesis, immune surveillance, and cancer progression. The ability of A3 enzymes to induce sublethal levels of mutations in HPV and extensive mutations in the host genome underscores their dual role in oncogenesis. Understanding this molecular interplay is critical for developing effective strategies to prevent and treat HPV-associated cancers. It is important to emphasize that most of the knowledge in this field has been derived from studies focusing on A3A, A3B, and A3C, particularly due to their nuclear localization, which is considered a relevant factor in HPV infection. Much less attention has been given to other A3 enzymes, and the role of natural A3 variations in HPV-associated cancers remains largely unexplored. While population-specific haplotypes and splice variants of A3 enzymes are known to have differential impacts on HIV-1, similar studies on HPV are currently lacking. Investigating whether these natural variations contribute to the differences in HPV prevalence across the globe would be an interesting area for future research.

### 2.2 Hepatitis B virus (HBV)

#### 2.2.1 HBV overview

HBV is a partially double-stranded DNA virus that belongs to the Hepadnaviridae family. It is classified into several genotypes (A to J), each with distinct geographic distributions and clinical outcomes. These genotypes are defined based on differences in the nucleotide sequence of the viral genome, with sequence divergence of more than 8% being used to distinguish between genotypes. The distribution of HBV genotypes varies by region, with genotype E being predominant in West Africa, including Ghana (Kafeero et al., 2023). Genotype variability has significant implications for disease progression, treatment response, and the development of liver cancer.

The epidemiological distribution of HBV is influenced by a variety of factors, including genotype variability, which plays a crucial role in determining the severity of the disease, the likelihood of complications, and the response to antiviral therapies. HBV infection remains a major global health challenge, with approximately 240 million people living with chronic HBV worldwide. This burden is particularly high in regions such as Africa (Kafeero et al., 2023) and the Asia-Pacific (Chen et al., 2000), where vaccination and surveillance efforts remain inadequate. In these regions, HBV infection is a leading cause of liver cancer, contributing significantly to morbidity and mortality.

**FIGURE 5:**
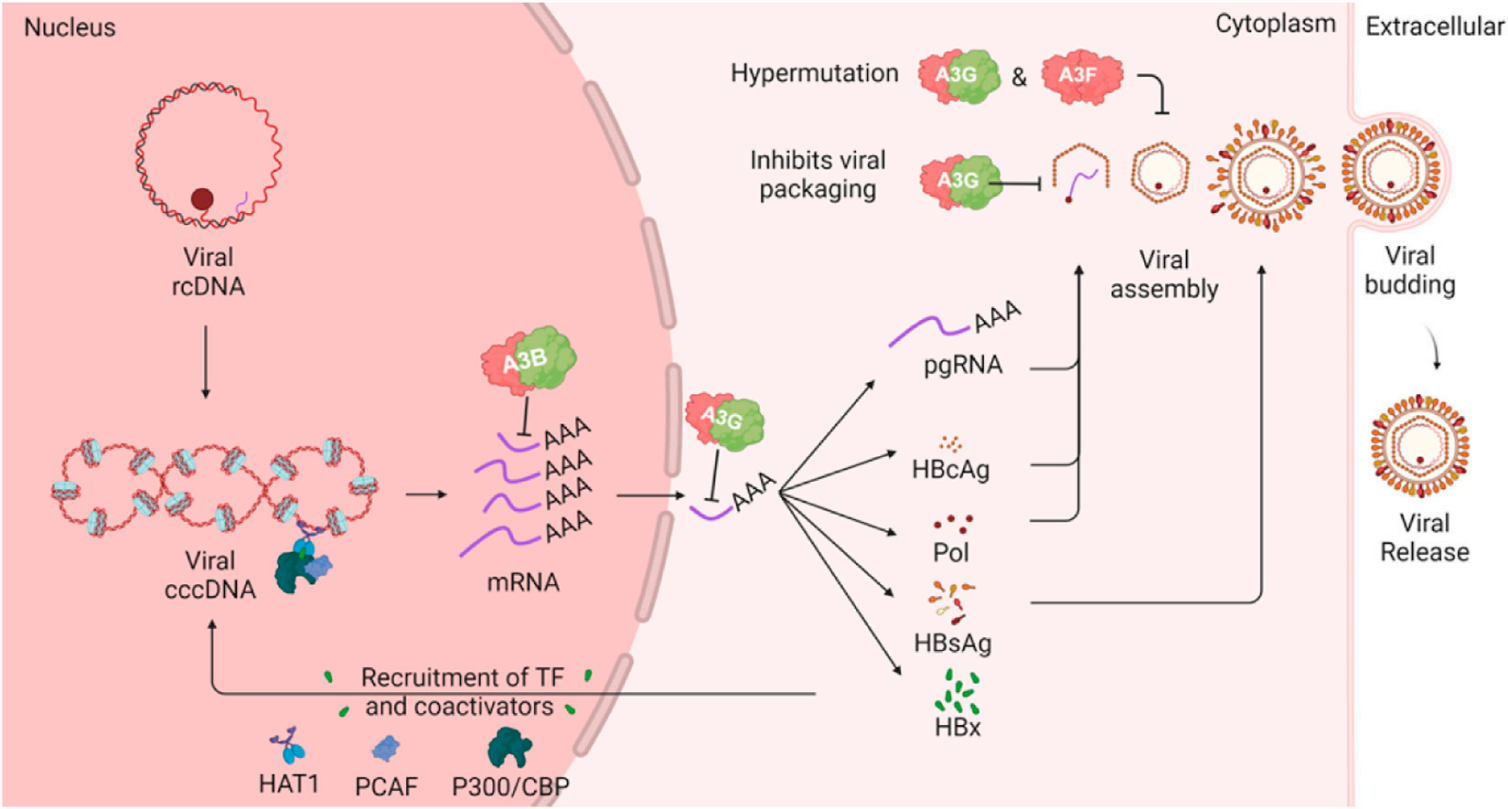
HBV lifecycle and interaction with A3 enzymes. The schematic illustrates how A3 proteins can target various steps in the HBV lifecycle. The cccDNA in the host nucleus encodes several viral mRNA products which can be targeted by A3B in the nucleus or by A3G in the cytoplasm. One of the viral mRNA products is pregenomic RNA (pgRNA), which is encapsulated by HBV polymerase. During this step, A3G can inhibit HBV through a deaminase-independent mechanism. Additionally, A3G and A3F, when incorporated into the viral nucleocapsid, can induce mutations in the HBV genome following reverse transcription of the pgRNA.

The replication of HBV involves a complex cycle that begins with the conversion of relaxed circular DNA (rcDNA) into covalently closed circular DNA (cccDNA) within the nucleus of hepatocytes (Figure 5). This cccDNA serves as the template for the transcription of viral RNAs, including pregenomic RNA (pgRNA), which is essential for the synthesis of viral proteins and the replication of the viral genome. HBV encodes several key proteins, including the surface antigen (HBsAg), core antigen (HBcAg), polymerase, and the X protein (HBx). The polymerase, or P protein, plays a central role in viral replication by functioning as a reverse transcriptase, DNA-dependent DNA polymerase, and RNase H, making it a critical target for antiviral therapies (Clark and Hu, 2015).

The HBx protein is a multifunctional oncoprotein that plays a critical role in the development of hepatocellular carcinoma by regulating cell cycle progression, signal transduction, and DNA repair. HBx also facilitates HBV replication by recruiting transcriptional coactivators such as CBP, P300, and PCAF to the cccDNA template enhancing viral gene expression and replication. Additionally, HBx modulates the host immune response, allowing HBV to evade immune surveillance and persist within the liver (Sartorius et al., 2020).

The ongoing replication of HBV within the host poses a significant risk for the development of liver cancer, particularly hepatocellular carcinoma (HCC). Current treatment strategies for HBV focus primarily on inhibiting viral replication, which reduces the risk of liver fibrosis and subsequent cancer development. However, a major limitation of these treatments is the persistence of cccDNA in the nucleus of infected cells, making complete eradication of HBV challenging. Recent research has highlighted the potential of A3B in targeting and degrading HBV cccDNA (Wang et al., 2023), offering a promising avenue for the complete elimination of the virus.

HBV can integrate its DNA into the host genome, leading to significant genomic instability, which is evident in liver cancer and other malignancies, such as diffuse large B-cell lymphoma, where HBV integration is frequently observed (Ren et al., 2018). The integration of HBV DNA into the host genome can profoundly impact gene expression, particularly when it occurs in critical regions such as gene promoters or untranslated regions. For example, integration events in the *TERT* gene, which encodes the catalytic subunit of telomerase, have been associated with increased expression of *TERT* in liver cancer samples, suggesting a direct link between viral integration and the activation of oncogenic pathways. Similarly, HBV integration in other genes such as *KMT2B* (Zhao et al., 2023) and *RGS12* (Yang et al., 2024) has been associated with the dysregulation of gene expression and the promotion of cancer cell proliferation. These findings underscore the importance of understanding the molecular consequences of HBV integration and its role in liver cancer development.

#### 2.2.2 A3 expression in HCC and chronic hepatitis B (CHB)

Several studies have compared the expression of A3 enzymes in normal and diseased liver tissues, yielding results that are often in consistent. For example, a study by Ni et al. (2020) shows that A3G has the highest expression in CHB patients, followed by liver cirrhosis patients and liver cancer patients. In contrast, in another study by Chen et al. (2010) found no significant difference in the level of A3G between CHB and HCC groups. Given the similarity in methods between these studies, the discrepancies could be attributed to population differences.

In HCC patients, several studies have consistently reported higher A3B expression in tumor tissues compared to normal tissues (Xu et al., 2007; Yang et al., 2015a; Luo et al., 2016). While A3D and A3F were also frequently overexpressed in liver tumors (Yang et al., 2015a; Yang et al., 2018; Luo et al., 2016), the expression of other A3 enzymes, such as A3A, A3C, and A3G, showed inconsistent patterns, with some studies reporting downregulation in cancers (Yang et al., 2015b; Luo et al., 2016).

HBV has also been shown to trigger the expression of APOBEC2 through miR-122 regulation, further contributing to the proliferation of liver cancer cells (Li et al., 2019). Overall, these studies identify A3B as the most consistently upregulated enzyme in HCC tumors, with variability observed in the expression of other A3 enzymes across different studies.

#### 2.2.3 A3 polymorphism in HCC

The polymorphisms G1896A pre-core and A1762T/G1764A basal core promoter (BCP) have been identified as risk factors for HCC. A study by Lau et al. reported that A3G protein levels are higher in wild-type HBV compared to the G1896A and A1762T/G1764A mutants, suggesting that the presence of HBV mutants may downregulate A3G expression. Despite these expression differences, the study found that A3G did not cause significant G-to-A hypermutations in the HBV cccDNA in a HepG2 cell model. This finding suggests that the increased A3G levels in wild-type HBV do not translate into higher mutational activity in the viral DNA (Lau et al., 2020)

Three SNPs rs2267398, rs2267401, rs2076109 combine to generate several *A3B* haplotypes. It has been shown that *A3B* haplotypes C-T-A, C-T-G, T-G-G, and T-T-G are associated with a lower risk of HCC compared to the reference haplotype. Additionally, the rs2267401-G allele has been found to significantly increase the risk of HCC when interacting with rs3890995-C allele of UDG. This combination has been shown to be associated with the generation of A3-induced mutations in the HBV genome and an increased risk of HCC (Liu et al., 2019).

#### 2.2.4 A3-induced HBV mutations in cancer

A3B, and depending on the study, other A3 enzymes, have been shown to be upregulated in tumor tissues of HCC patients (Xu et al., 2007; Yang et al., 2015a; Luo et al., 2016). The mutations exerted by the A3 enzymes in the HBV genome are more abundant in tumor biopsies compared to non-tumor samples, suggesting a role in promoting carcinogenesis. For instance, one study reported that the frequency of A3-induced mutations in cancer samples is 4.85 per 1,000 sites, compared to only 0.16 per 1,000 sites in non-tumor samples (Zhang et al., 2021). These mutations may serve as a driving factor in the development of liver cancer by altering the viral genome to promote the survival and proliferation of neoplastic cells.

The mutations induced by A3 enzymes, particularly in the *HBx* gene, are thought to enhance the colony-forming ability and proliferative capacity of neoplastic cells (Shukla and Kumar, 2012). For example, G-to-A mutations in the *HBx* gene have been linked to the creation of C-terminally truncated mutants, which are associated with increased oncogenic potential in liver cells (Zhang et al., 2021; Agustiningsih et al., 2024).

A3-induced *HBx* mutations have also been observed in preneoplastic liver samples from CHB patients, suggesting that A3-induced mutation of HBV may play a role in the early stages of liver cancer development, providing a selective growth advantage to preneoplastic and neoplastic hepatocytes (Zhang et al., 2021).

Recent studies have demonstrated that serum HBV mutations, particularly those related to A3 enzymes, can serve as predictive markers for HCC occurrence and prognosis. The HBV Quasispecies Complexity (QC), a measure of viral genetic diversity, was shown to be positively correlated with A3 expression levels, being higher in adjacent normal tissues compared to tumors, and was associated with early tumor stages. Notably, HBV was reported to evolve more rapidly in the sera than in tumors, and mutations such as A1762T/G1764A showed contrasting prognostic implications depending on their location. High QC and frequent mutations in the sera were linked to poorer overall survival and recurrence-free survival (RFS), whereas in tumors, they predicted improved RFS (Yin et al., 2021).

### 2.3 Herpesvirus

#### 2.3.1 Herpesvirus overview

There are nine known herpesviruses that infect humans, belonging to the Herpesviridae family, which is divided into three subfamilies: Alphaherpesviruses, Betaherpesviruses, and Gammaherpesviruses (Arvin et al., 2007). Herpes Simplex Virus types 1 and 2 (HSV-1 and HSV-2) and Varicella-Zoster Virus (VZV) belong to the Alphaherpesvirus subfamily. HSV-1 primarily causes oral herpes but can also cause genital herpes, while HSV-2 more often causes genital herpes. VZV is responsible for chickenpox (varicella) and shingles (herpes zoster).

Cytomegalovirus (CMV), Human Herpesviruses 6A, 6B, and 7 (HHV-6A, HHV-6B, and HHV-7) belong to the Betaherpesvirus subfamily. CMV can cause a variety of diseases, particularly in immunocompromised individuals. HHV-6A is less common and has been associated with neurological diseases, while. HHV-6B causes roseola infantum, a common childhood illness. HHV-7, like HHV-6B, also causes roseola and other similar conditions.

Finally, Epstein-Barr Virus (EBV) and Human Herpesvirus 8 (HHV-8), also known as Kaposi’s sarcoma-associated herpesvirus (KSHV), belong to the Gammaherpesvirus subfamily. EBV causes infectious mononucleosis (mono) and is associated with certain types of cancer, such as Burkitt’s lymphoma and nasopharyngeal carcinoma. KSHV, on the other hand, is linked to Kaposi’s sarcoma, a type of cancer commonly seen in AIDS patients. Herpesvirus infections are lifelong and incurable, with the virus establishing latency in host cells and potentially reactivating to produce new infections (Carneiro et al., 2022).

Herpesviruses possess linear double-stranded DNA genomes, ranging from 120 to 240 kilobases, encoding approximately 80–250 distinct proteins. The viral particle, tmeasuring approximately 100–200 nm in size, consists of an icosahedral capsid, a tegument layer containing viral proteins and RNAs essential for genome replication, and a lipid bilayer envelope with glycoproteins for cell entry (Cheng et al., 2021). A significant characteristic of herpesviruses is their ability to switch between latency and lytic replication. During latency, the viral genome circularizes in the host cell nucleus, disguising as cellular DNA through the incorporation of epigenetic modifications such as methylation, histone modification, and chromatinization, thereby minimizing viral gene expression to evade immune detection. Lytic replication, which can be triggered by environmental factors such as stress or immunosuppression, involves coordinated expression of viral genes necessary for DNA replication, particle assembly, and virion release (Brown, 2017). Despite their high-fidelity DNA replication, herpesviruses can still accumulate mutations, though these are significantly less frequent compared to RNA viruses, which have high mutation rates due to error-prone polymerases. Although herpesvirus genomes interact with A3 enzymes, they do not typically exhibit C/G-to-T/A mutations characteristic of A3 editing, likely due to chromatinization and precise DNA replication mechanisms (Weller and Coen, 2012).

With respect to A3 enzymes, EBV is one of the most studied herpesviruses. EBV infects approximately 95% of the adult global population, with varying prevalence across age groups and socio-economic backgrounds. For example, in the United States, infection rates range from 54% among younger children to over 82% in older adolescents. Transmission typically occurs through oral secretions, with primary infection predominantly occurring in early childhood in less affluent regions, whereas in more developed areas, it often happens during adolescence (Hammerschmidt and Sugden, 2013). EBV has a ∼173 kilobase double-stranded DNA genome, which encodes 100 protein-coding genes along with non-coding RNAs and microRNAs. This genetic makeup facilitates a dual life cycle comprising latent and lytic phases. The latent phase involves limited expression of viral genes, including six EBV nuclear antigens, three latent membrane proteins, and two small RNAs, with latent membrane protein 1 (LMP1) playing a key role as an oncoprotein (Murata et al., 2021). During latency, EBV establishes long-term infection primarily in B lymphocytes, while engaging in active replication within the oral mucosal epithelium. The virus’s ability to infect different cell types, such as B lymphocytes and epithelial cells, is facilitated by specific viral proteins like gp350/220 and gp42 for B cell entry, and BMRF2 for interaction with β1 integrin in epithelial cell entry. Additionally, EBV exploits the ephrin receptor A2 (EphA2) as an entry pathway into epithelial cells, demonstrating its adaptability in using multiple receptors and entry strategies (Ali et al., 2015).

#### 2.3.2 Interaction between herpesvirus and A3 enzymes

**FIGURE 6:**
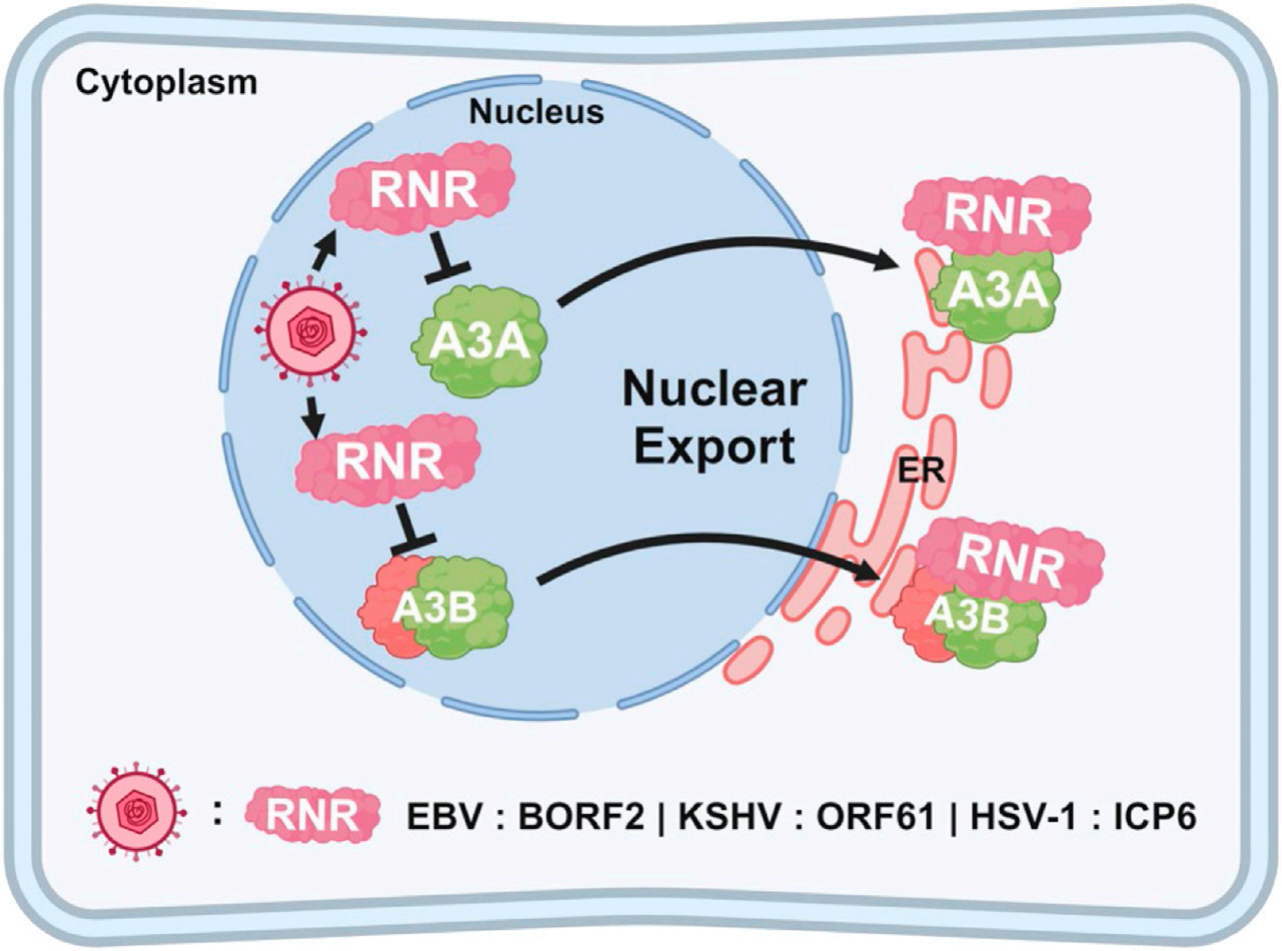
Relocalization of A3 enzymes by herpesviruses. The schematic illustrates how herpesviruses (EBV, KSHV, and HSV-1) evade nuclear A3 proteins by expressing RNR proteins (BORF2, ORF61, and ICP6, respectively), which target A3 enzymes for relocalization. Once exported out of the nucleus, the A3 enzymes are aggregated, packaged into autophagosomes, and selectively degraded through aggrephagy.

While A3 enzymes have been well-studied in the context of retroviruses like HIV-1, more recent studies have highlighted their interaction with herpesviruses, albeit with limited impact on viral replication (Moraes et al., 2022). Evidence suggests that EBV, KSHV, and HSV-1 may have developed mechanisms to evade A3-mediated restriction (Moraes et al., 2022). Unlike retroviruses, herpesviruses have low mutation rates, indicating they are less susceptible to deamination-dependent inhibition by A3 enzymes. However, studies have shown that these viruses use their ribonucleotide reductase (RNR) large subunit to interact with and inhibit A3 enzymes, particularly A3B and A3A (Figure 6). Specifically, the RNR large subunit from EBV (BORF2), KSHV (ORF61), and HSV-1 (ICP6) has been shown to bind to these two A3 enzymes, inhibiting their catalytic activity and relocalizing them from the nucleus to cytoplasmic aggregates (Cheng et al., 2021). This interaction prevents the A3 enzymes from accessing viral DNA during replication, thereby protecting the viral genome from C-to-U mutations.

The interaction between EBV BORF2 and A3B is particularly well understood. BORF2 specifically binds to the C-terminal catalytic domain of A3B, which is crucial for its DNA deaminase activity. This binding effectively inhibits A3B by preventing its interaction with viral ssDNA, thereby blocking its mutagenic potential. However, it is noteworthy that the deletion of BORF2 from the EBV genome does not impair viral replication (Cheng et al., 2018), raising important questions about the role of BORF2-mediated relocalization of A3A and A3B as a definitive viral counter-defense mechanism. Despite this, the conserved interaction between herpesvirus RNRs and A3 enzymes, coupled with evidence of positive selection in both A3B and BORF2, has led to the hypothesis to that an ongoing evolutionary arms race exists between these viral and host factors. This dynamic interaction is believed to have driven the development of these viral countermeasures (Cheng et al., 2019).

#### 2.3.3 Role of A3 enzymes in herpesvirus-associated cancers

Recent studies have highlighted an interaction between herpesvirus RNRs and A3B/A enzymes (Figure 6), yet the implications of this interaction in the development and progression of herpesvirus-associated cancers remain poorly understood. One study focusing on gastric cancers identified a positive correlation between the mRNA expression levels of A3 enzymes and A3-induced mutations in EBV-positive tumors, suggesting that A3 upregulation in these cancers could contribute to an increased rate of DNA mutation (Bobrovnitchaia et al., 2020).

Another study demonstrated that the EBV latent membrane protein 1 (LMP1) induces the expression of A3B and A3F in nasopharyngeal cells and found a positive correlation between LMP1 and A3B expression in nasopharyngeal cancer patients. This study also reported significant hypermutation in mitochondrial DNA in cells expressing LMP1 and showed that higher levels of LMP1 and A3B correlated with neck metastasis in patients. The hypermutation of mitochondrial DNA induced by A3B was hypothesized to promote metastasis in these cancers (Wakae et al., 2020).

A study by Jung et al. (2018) investigated the role of L1 retrotransposons in gastrointestinal cancers, which are typically suppressed by DNA methylation and immune defenses. In cancer, however, these suppression mechanisms can become disrupted, allowing L1 retrotransposition and driving genomic instability. The study showed that tumors displaying high immune activity, particularly those associated with EBV infections, had fewer L1 insertions. This finding suggests that a strong immune response, potentially influenced by viral presence, can maintain genomic stability by actively suppressing L1 activity (Jung et al., 2018).

In the context of therapeutic interventions, a clinical study has explored the potential of bortezomib combined with ifosfamide, carboplatin, and etoposide in inducing lytic activation of gammaherpesviruses in HIV-positive lymphomas. This strategy, potentially mediated by the preservation of cytidine deaminase A3G, showed promising outcomes in controlling lymphoma progression. These findings illustrate a novel approach to harnessing herpesvirus biology for clinical benefit (Reid et al., 2018).

### 2.4 Polyomaviruses

#### 2.4.1 Polyomavirus overview

Human polyomaviruses (HPyVs) are a group of non-enveloped viruses with, a circular dssDNA genome. These viruses are classified into different genera, with the most significant for human health being the Alphapolyomavirus and Betapolyomavirus genera. Among these, notable types include BK polyomavirus (BKPyV), John Cunningham polyomavirus (JCPyV), Merkel cell polyomavirus (MCPyV), and Trichodysplasia spinulosa polyomavirus (TSPyV) (Bauman et al., 2011; Lee et al., 2011). Although these viruses are very common, they are generally harmless and remain latent within the human body except in immunocompromised individuals (Prado et al., 2018).

HPyVs target various cell types depending on the virus. BKPyV mainly infects renal epithelial cells, while JCPyV targets glial cells in the brain. MCPyV infects Merkel cells in the skin, and TSPyV targets hair follicle cells. The diversity in cell tropism reflects the variety of diseases these viruses can cause (Prado et al., 2018).

BKPyV is found in about 80% of the global population, usually acquired during childhood without causing symptoms. However, in kidney transplant patients, using immunosuppressive drugs to prevent organ rejection, BKPyV can reactivate and lead to nephropathy, a serious condition that can compromise the transplanted kidney. The prevalence of BKPyV-associated nephropathy varies, affecting between 1% and 10% of kidney transplant recipients worldwide (Rinaldo et al., 2013).

JCPyV is another widespread virus, with an estimated 50%–80% of the adult population haboring it. JCPyV also typically remains dormant in healthy individuals but can reactivate in immunocompromised individuals, particularly those with AIDS or undergoing immunosuppressive therapies. When reactivated, JCPyV can lead to progressive multifocal leukoencephalopathy (PML), a rare but devastating brain disease that is almost universally fatal without treatment. The global incidence of PML is challenging to estimate due to its rarity, but it is a significant concern for populations at risk, especially in areas with high rates of HIV/AIDS (Okada et al., 2001; Mormando et al., 2021).

MCPyV, discovered more recently in 2008, is particularly intriguing because of its clear link to cancer. MCPyV is found in approximately 80% of Merkel cell carcinomas (MCC), a rare but aggressive form of skin cancer that primarily affects older adults and immunosuppressed individuals. The incidence of MCC is highest in regions with large elderly populations, such as Northern Europe, Australia, and the United States, with an estimated 2,500 new cases each year in the U.S. alone. Although rare, MCC is highly aggressive, with a 5-year survival rate of around 60% if detected early but dropping significantly if diagnosed at a later stage (IARC Working Group on the Evaluation of Carcinogenic Risks to Human, 2014).

TSPyV is the etiological agent behind Trichodysplasia spinulosa, a rare skin condition characterized by the development of spiny papules. TSPyV infection is generally benign, but it can lead to more severe skin manifestations in immunosuppressed individuals. This condition is much less common than the cancers associated with other HPyVs, and global data on its prevalence is limited due to its rarity (Curman et al., 2021).

The HPyV genome is organized into three regions: the early region, the late region, and the noncoding control region. The early region is particularly important because it encodes the large T (LT) antigen and small T (sT) antigen, which are critical for viral replication and can disrupt normal cellular functions, leading to uncontrolled cell growth and, ultimately, cancer (Howes et al., 1996). For instance, MCPyV, when integrated into the host genome, expresses a truncated form of the LT antigen that retains the ability to inactivate key tumor suppressor proteins like pRb but loses the capacity to initiate viral replication. This mechanism enables the virus to drive the development of MCC without killing the host cells (Delbue et al., 2012).

Polyomaviruses interact intricately with various host enzymes to manipulate cellular processes and create a favorable environment for viral replication and persistence. One of the most significant interactions occurs between polyomaviruses and the cellular enzyme family of cyclin-dependent kinases (CDKs). The LT antigen, a key protein encoded by the early region of the polyomavirus genome, interacts directly with CDKs, particularly CDK2, to drive the host cell into the S phase of the cell cycle. This manipulation ensures that the cellular machinery required for DNA replication becomes available, allowing the virus to hijack these processes to replicate its own DNA (Neu et al., 2009; Justice et al., 2015).

Another critical interaction involves the phosphatase enzyme PP2A, which is targeted by the sT antigen of polyomaviruses. PP2A is a major regulator of multiple signaling pathways within the cell, including those controlling cell growth, division, and apoptosis. The binding of sT antigen to PP2A disrupts its normal function, leading to the activation of pathways that promote cell survival and proliferation (Ingebritsen and Cohen, 1983; Pallas et al., 1990).

Induction of A3 enzymes by polyomaviruses, their interaction with the viral LT antigen and their association with tumor mutagenesis have also been reported in several studies (Neu et al., 2009), as discussed below.

#### 2.4.2 Interaction between polyomaviruses and A3 enzymes

A study by Verhalen et al. (2016) demonstrated that A3B is specifically upregulated by BKPyV infection in primary kidney cells, and that the upregulated enzyme is active. Additionally, the BKPyV LT antigen, as well as LT antigens from related polyomaviruses, is sufficient to upregulate A3B expression. Interestingly, although the specific knockdown of A3B showed minimal short-term effects on productive BKPyV infection, the preferred target sequences of A3B appear to be depleted in BKPyV genomes, possibly suggesting a long-term influence of A3 enzymes on viral sequence composition. These findings have led to speculation that increased A3B activity may contribute to PyV-mediated tumorigenesis and influence viral sequence composition over extended evolutionary periods (Verhalen et al., 2016).

A computational study by Shapiro et al. (2021) highlights the role of A3A and A3B in HPyV mutagenesis. It shows that early-expressed genes, particularly LT antigen, are more prone to A3-induced mutations due to their expression at a stage when the viral genome is more accessible to these enzymes. By contrast, late genes such as those encoding viral capsid proteins (VP1, VP2, VP3) are less affected by A3-induced mutations. Additionally, the authors propose that polyomaviruses may have evolved to reduce the number of A3-targeted motifs, particularly in the LT antigen gene, to evade these mutations. The lower representation of A3 hotspot TC in the early genes compared to late genes are provided as supporting evidence (Shapiro et al., 2021).

An *in vitro* study using a normal human urothelium model indicates that BKPyV infection significantly increases the level of A3A and A3B proteins and causes damage in urothelium cells, likely due to A3 deaminase activities (Baker et al., 2022). Indeed, the BKPyV’s LT antigen has been shown to induce the expression of A3B through a mechanism involving the inhibition of retinoblastoma protein (pRb) by LT antigen, which leads to cell cycle re-entry and activation of the E2F1 transcription factor, thereby upregulating A3B expression (Starrett et al., 2019). It is thought that the single-stranded DNA displacement loops formed during BKPyV infection could serve as substrates for A3 enzymes. This interaction results in increased deaminase activity and leads to more abasic sites in the host genome, indicative of DNA damage (Baker et al., 2022). Indeed, a significant correlation between the A3B protein level and the extent of DNA damage has been reported (Verhalen et al., 2016). Furthermore, proximity ligation assays demonstrated that A3 proteins were frequently found in close proximity to LT antigen within the nucleus of infected urothelial cells, suggesting that LT antigen might play a role in facilitating the recruitment of A3 enzymes to the host genome during BKPyV infection (Baker et al., 2022).

In contrast to the study by Baker et al. (2022), which shows the upregulation of both A3A and A3B, Rao et al. (2023) showed that BKPyV infection in the HBLAK cell line leads to a significant upregulation of A3B but not A3A. This upregulation was observed early after infection and persisted over time, suggesting that BKPyV infection has the potential to induce A3B-mediated mutagenesis in tumors (Rao et al., 2023).

#### 2.4.3 Role of A3-polyomavirus interaction in cancer

*FGFR3* and *PIK3CA* genes are frequently mutated in bladder cancer, especially in non-muscle-invasive bladder cancer (NMIBC). Specific mutations within the proteins expressed by these genes, particularly *FGFR3-S249C*, *PIK3CA-E542K/E545K*, have been associated with the activity of A3 enzymes, particularly A3A and A3B (Kompier et al., 2010; Rao et al., 2023). A detailed analysis of these mutations across numerous bladder cancer cases indicates that they occur more commonly in never-smokers compared to ever-smokers, suggesting that these mutations may arise independently of smoking-related carcinogens (Kompier et al., 2010). These data have led to the hypothesis that viral infections might contribute to the enrichment of A3-induced driver mutations in tumors of never-smokers. To investigate this hypothesis, the authors conducted RNA sequencing (RNA-seq) on bladder tumors, revealing a strong association between BKPyV positivity and the presence of A3-induced mutations in NMIBC tumors. Further analysis indicated that BKPyV-positive NMIBC tumors harboring *FGFR3* or *PIK3CA* mutations exhibited a higher likelihood of progressing to muscle-invasive bladder cancer (MIBC) compared to BKPyV-negative tumors. These findings suggest that BKPyV infection may not only drive the initial development of these mutations but also play a role in disease progression.

Interestingly, the study also highlighted distinct patterns of BKPyV infection in NMIBC and MIBC. BKPyV-positive NMIBC tumors exhibited a different viral expression pattern compared to the single MIBC tumor with BKPyV integration. This suggests that episomal BKPyV (non-integrated) is more common in NMIBC, while integrated BKPyV might be associated with more advanced disease.

Another study analyzing numerous MCC samples showed that A3 proteins, particularly A3H, A3G, and A3A, are expressed in a significant portion of MCPyV-positive MCC tumors, with their expression correlating with viral LT expression. Additionally, the study identified a strong A3-induced mutational signature in the MCPyV LT antigen in MCC, suggesting that A3 enzymes likely contribute to the formation of premature stop codons and truncation of the LT antigen in virus-positive MCCs. Mutations in the LT antigen were enriched in specific A3 hotspots, pointing to the potential involvement of all A3 enzymes except A3G (Soikkeli et al., 2022).

## 3 Current knowledge gaps and future directions

### 3.1 A3-targeted immunotherapy

Dysregulation of A3 enzymes, particularly in response to viral infections, can be leveraged to develop targeted immunotherapies. Indeed, studies have shown that tumors with high levels of A3-induced mutations respond better to immune checkpoint inhibitors (ICIs) and targeted immunotherapies (Miao et al., 2018; Litchfield et al., 2021). This improved responsiveness is attributed to the increased tumor mutational burden resulting from A3-mediated mutagenesis, which generates neoantigens recognizable by the immune system. For instance, in non-small cell lung cancer (NSCLC), upregulation A3B correlates positively with immunotherapy response biomarkers, including PD-L1 expression and T-cell infiltration (Wang et al., 2018). Notably, the A3-induced mutational signature is enriched in NSCLC patients who exhibit durable clinical benefits after immunotherapy.

In murine mammary tumor models, A3 activity induces antitumor adaptive immune responses and sensitizes HER2-driven tumors to anti-CTLA-4 checkpoint inhibition (DiMarco et al., 2022). Additionally, another study used A3B to generate heteroclitic neoepitopes in unmodified “vaccine tumors” (Driscoll et al., 2020). These neoepitopes activate *de novo* T cell responses that can cross-react with unmodified tumors, leading to high cure rates in both subcutaneous and intra-cranial tumor models. Similarly, in breast cancer, A3 activity has been shown to enhance immune infiltration and induce antitumor adaptive immune responses, sensitizing HER2-driven tumors to anti-CTLA-4 checkpoint inhibition (DiMarco et al., 2022).

Despite these promising advancements, significant knowledge gaps remain regarding the precise mechanisms by which these mutations contribute to neoantigen formation and immune recognition. Furthermore, the potential risks associated with enhancing A3 activity, such as increased genomic instability, require careful evaluation before A3-targeted therapies can be advanced to clinical trials.

### 3.2 The “hit-and-run” hypothesis

**FIGURE 7:**
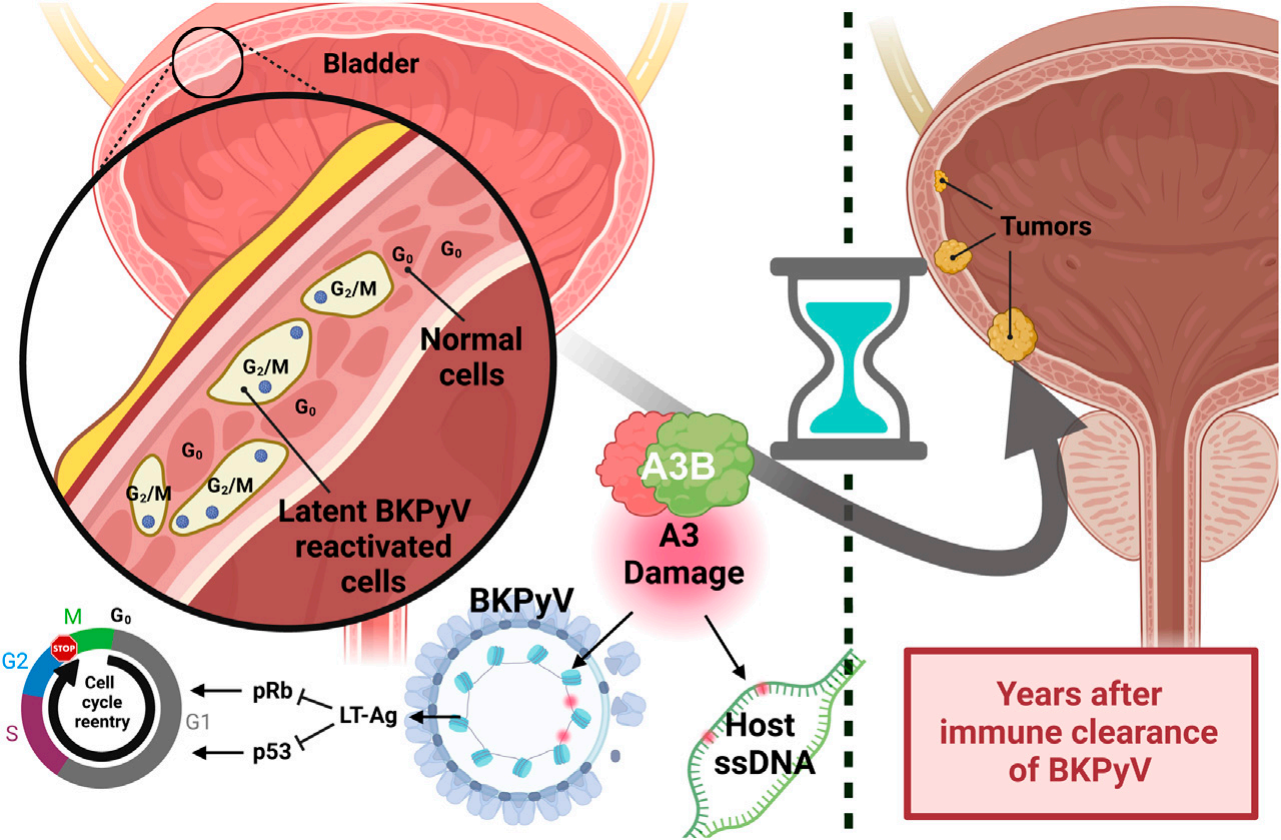
Hit-and-run carcinogenesis caused by reactivation of papillomavirus. The schematic illustrates the proposed link between papillomavirus reactivation and the development of bladder cancer years later. Reactivation of BKPyV in urothelial cells results in the production of the LT antigen, which inhibits the host’s pRb and p53 proteins. The inhibition causes urothelial cells to re-enter the cell cycle and improperly progress through the G2/M checkpoint. In response, A3B activity acts to clear the papillomavirus infection but also induces genomic mutations. Over time, the expansion of these mutated cells can lead to tumor formation.

Currently, around 13%–18% of cancer cases are attributed to viral infection. However, the “hit-and-run” hypothesis suggests that this number is likely an underestimate. According to this hypothesis, the virus can cause an initial oncogenic damage that results in tumor mutations, predisposing cells to cancer development long after the infection has been resolved by the immune system (Figure 7). This hypothesis may explain why a clear link between viral infections and A3-induced mutagenesis has not been established yet.

Evidence supporting this hypothesis has emerged through limited studies of oncogenic viruses. For instance, adenoviruses have been shown to induce tumorigenesis in host cells that persist even after the virus is no longer detectable (Nevels et al., 2001). HPV, commonly associated with cervical and other cancers, also supports the hit-and-run model. While HPV DNA is often found in early-stage tumors, the virus sometimes disappears in advanced cancers, suggesting it may initiate tumorigenesis without being required for tumor maintenance (Narisawa-Saito and Kiyono, 2007). Similarly, HBV has been shown to integrate into the host genome, causing lasting genetic and epigenetic changes even after the virus has been cleared. This genomic integration can lead to cellular alterations that promote oncogenesis, consistent with the hit-and-run mechanism (Hoppe-Seyler et al., 2018). EBV offers additional support for this model, as it induces early oncogenic changes in infected cells. Even if the viral genome is undetectable at later cancer stages, damage initiated during early infection may persist, contributing to cancer development (Niller et al., 2011).

Despite these examples, direct proof of hit-and-run oncogenesis remains elusive due to the lack of evidence linking transient viral presence to cancer development. Traditional causal criteria, such as Koch’s postulates, are not fully applicable in these cases because the virus may no longer be detectable when cancer symptoms arise (Casadevall and Pirofski, 2003). However, advances in high-throughput sequencing technologies are providing new opportunities for studying viral sequences in pre-cancerous and early neoplastic tissues. These methods may enable the detection of transient viral sequences present during the early stages of tumorigenesis that contribute to cancer onset.

### 3.3 Tumor vs. tumor microenvironment (TME)

To understand the role of A3 enzymes in cancer development, it is crucial to recognize their expression across cell types, including immune cells, tumor cells, and normal cells within the TME. This broad expression poses significant challenges in identifying the specific contributions of A3 enzymes from different cell types, particularly when bulk sequencing methods are used. To fully understand the role of viral infection in A3 dysregulation, future studies should focus on single-cell resolution profiling of these enzymes. Single-cell sequencing techniques provide a promising avenue for identifying cell-type-specific *A3* expression patterns (Zhang et al., 2023), offering a high-resolution view of *A3* dynamics across different cell populations and revealing changes induced by viral infection. Complementing this approach, spatial transcriptomics can map *A3* expressions within tumors and surrounding tissues, offering insights into the spatial distribution of *A3*s influenced by viral infection. Indeed, recent improvements in single-cell data analysis tools are enabling the detection of viral reads at the single-cell level (Whitmore et al., 2024). *In situ* hybridization techniques offer another method to visualize *A3* mRNA expression in different cell types within the tumor and TME, allowing for direct observation of *A3* expression patterns in the context of tissue architecture. Additionally, developing more specific A3 antibodies for immunohistochemistry would enable precise localization of A3 proteins within tissue sections, further enhancing our understanding of A3 distribution and the changes induced by viral infections.

High-resolution profiling of A3 enzymes has the potential to identify therapeutic strategies that specifically target viral-induced A3 dysregulation in tumor cells while minimizing impact on surrounding non-tumor cells. Ultimately, this comprehensive analysis of A3 activity within tumors and their microenvironment could inform the development of more effective and targeted approaches for cancer treatment and prevention.

### 3.4 A3 variations

*A3* polymorphisms and alternative splicing can significantly influence cancer susceptibility and viral resistance, with notable geographical variations. Investigating the functional differences among the A3 variants can shed light on the host and viral determinants of cancer disparities across populations, underscoring the importance of considering A3 genetic diversity in cancer risk assessment and treatment strategies.

A significant knowledge gap in the field is that, with few exceptions, A3 studies have focused on viral infection and cancer independently, rather than exploring their combined molecular interplay. The complex interactions between A3 enzymes and viral infections in the context of cancer remain a largely unexplored. For instance, there is a 29.5 kb germline deletion polymorphism within the A3 locus, which deletes the 3′end of the A3A gene and most of the A3B gene, resulting in a hybrid gene that produces mRNA combining A3A’s coding region with A3B’s untranslated region (UTR). This deletion is associated with higher A3A mRNA stability, elevated A3 levels, and increased rates of DNA mutation, correlating with a higher cancer risk (Long et al., 2013; Gansmo et al., 2018; Pan et al., 2021). The prevalence of this deletion varies widely across populations, occurring in 37% of Asians, 6% of Europeans, and 57.7% of American descendants. A study of NSCLC in the southern Chinese population found that individuals with the homozygous deletion had a 2.71-fold higher risk of developing NSCLC compared to those without the deletion genotype (Ben et al., 2021), suggesting that the A3B deletion may serve as a potential biomarker for early-stage NSCLC screening in populations with high deletion frequency.

A study by Rouf Banday et al. (2021) examined several A3A and A3B isoforms, revealing that canonical isoforms are more mutagenic than others. Notably, the expression level of the canonical A3B isoform associated with shorter progression-free survival in bladder cancer (Rouf Banday et al., 2021).

A3H is particularly polymorphic within the A3 family, displaying four major haplotypes (I–IV) and three major splice variants (SV182, SV183, and SV200). These A3H variants exhibit population-specific distributions and distinct stability and activity profiles (Sadeghpour et al., 2021). For instance, European and Asian populations have higher frequencies of the relatively unstable A3H haplotype I, while African populations have higher frequencies of the stable A3H haplotype II. Among the main A3H haplotypes, only haplotype I, which encodes a less stable protein with minimal antiviral activity, has been implicated as a potential tumor mutagen, though its contribution to tumor mutagenesis is likely minor (Carpenter et al., 2023). Conversely, A3H haplotype II is a potent viral mutator but has not been linked to tumor mutagenesis. Interestingly, this haplotype also produces a unique splice variant (SV200), which is cleaved by the HIV-1 protease. Whether these extensive genomic, transcriptomic, and virus-host interaction differences among A3H variants contribute to population-specific variations in innate immunity against retroviruses and potentially influence cancer susceptibility, remains an open question.

While these examples highlight the diversity of A3 enzymes and the complexity of the complexity of their biology, much remains uknown about how viral infections differentially impact tumor initiation, mutagenesis, and progression in individuals with different A3 variants.

## 4 Discussion

A3 enzymes play a critical role in protecting the host from viral infection. However, these proteins also act as a double-edged sword; when chronically dysregulated by viral infections, they can promote the development and progression of various cancer types. The intricate relationship between viral infections, A3 dysregulation, and cancer development has become increasingly evident in recent years. A3-induced mutational signatures are particularly prevalent in cervical cancer, bladder cancer, and head and neck cancer, many of which have known viral origins (Burns et al., 2013; Roberts et al., 2013; Alexandrov et al., 2020; Baker et al., 2022). This pattern suggests that certain viruses may contribute to host DNA mutations by upregulating A3 enzymes, which in turn drive the mutagenic activity observed in these cancers. To delineate the scope of this dysregulation across the cancer landscape we summarized studies examining the interplay between specific viral infections and their association with individual cancers.

Notably, A3-induced mutational signatures are not confined to virus-associated cancers; they are also present in several other cancers, such as lung and breast cancers (Alexandrov et al., 2020), for which a direct viral association has not been established. This observation implies that other mechanisms such as inflammation, DNA damage response, hypoxia, and interferon signaling, can also induce A3 activity (Butler and Banday, 2023). Alternatively, as-yet-undiscovered viral agents could be involved. Of note are certain kidney cancers that exhibit A3-induced mutational signatures. Emerging evidence suggests that polyomaviruses, such as Merkel cell polyomavirus and BK virus, may play a role in modulating A3 enzyme activity in these cancers (Starrett et al., 2019; Baker et al., 2022; Soikkeli et al., 2022). These observations highlight the need for further research to investigate the viral origins of cancers, especially in cases where the connection between viral infection and cancer development remains unclear.

A significant challenge in this field is the conflicting evidence regarding which A3 enzymes are relevant in which virus-associated cancers. One reason for this inconsistency could be that many studies rely on overall expression levels of A3 enzymes in tumor tissue biopsies, which include both tumor and non-tumor cells, including immune cells. Since different immune cells express various A3 enzymes (e.g., B cells express A3B and dendritic cells and macrophages express A3A), high levels of A3A in a tumor biopsy, for example, may not necessarily indicate upregulation in tumor cells. Instead, it could reflect increased infiltration of dendritic cells and macrophages in the tumor tissue. Therefore, these mixed expression signals must be carefully deconvoluted at the single-cell level to accurately associate specific A3 enzymes with viral infection and tumor mutagenesis.

Importantly, even if this expression signal deconvolution is successfully performed, another complication arises from the potentially transient or episodic expression of A3 enzymes in tumors (Petljak et al., 2019). For example, viral infection might initially upregulate A3A, leading to DNA mutations, but by the time tumor samples are collected, A3A expression in tumor cells could return to baseline levels. This transient expression could obscure the true relationship between A3 enzyme activity and cancer progression.

As described earlier, the “hit and run” hypothesis further complicates efforts to establish a clear connection between viral infection and cancer. If the virus that triggered the initial A3 dysregulation has been cleared from the tumor, simply relying on viral sequence reads to determine the virus positivity of cancers may not be sufficient. This limitation hampers our ability to confidently classify samples as virus-positive or virus-negative and draw reliable conclusions about the sources of dysregulated processes in cancer, as well as their association with patient survival.

Finally, while recent research has significantly advanced our understanding of virus-induced A3 dysregulation in cancer, there is still much to learn about how natural variations in the A3 locus (Duggal et al., 2013; Sadeghpour et al., 2021), as well as geographic differences in virus variants (Mehanna et al., 2016), influence these processes at both the population and individual levels. To effectively incorporate these interactions into future precision medicine approaches, it will be crucial to gain a deeper understanding of the impact of these natural variations.

Moving forward, studies must focus on unraveling the multifaceted interplay between viruses and A3 enzymes across different cancers, considering the factors described above including the “hit and run” theory, episodic A3 expression, natural variations in the A3 locus and viral genomes, and the need for more advanced bioinformatic tools to separate the expression profiles of tumor and non-tumor cells. Additionally, there is a need to explore the potential involvement of other, currently unidentified viruses in cancers where A3-induced mutational signatures are present but no clear viral association has been established. These efforts will be crucial in advancing our understanding of the viral origins of cancer and in developing more effective prevention and treatment strategies.
